# Supplementary material for: Anti-Obesity Medications and the Risk of Obesity-Related Cancers in Older Women: A Propensity Score Matching Analysis of 2007–2015 SEER-Medicare Data
Source: Cancers (Basel). 2025 May 11;17(10):1624. doi: 10.3390/cancers17101624 (PMC12109998; doi:10.3390/cancers17101624)
Supplement: Supplementary file 1 [file cancers-17-01624-s001.zip › cancers-3582253-supplementary.pdf]

Supplementary Materials

Table S1. Variance Inflation Factor (VIF) for multicollinearity checks among predictors.

Table S2. Baseline characteristics according to the use of phentermine before and after 1:2 propensity score matching.

Table S3. Baseline characteristics according to the use of liraglutide before and after 1:2 propensity score matching.

Figure S1. Non-parametric Kaplan–Meier estimates of time to incident ORCs for women ≥65 years old stratified by the pre-diagnostic use of AOM in 1:2 propensity score matched cohort from 2007-2015 SEER-Medicare data.

Table S4. HRs and 95% CIs for the association between use of AOM and risk of ORCs (BrCa, CRC, ECa, and OCa) among women aged ≥65 years with and without diabetes, in a 1:2 propensity score matched cohort.

Table S5. Subdistribution hazard ratios (sHRs) and 95% CIs for the association between use of AOM and risk of ORCs (BrCa, CRC, ECa, and OCa) among women aged ≥65 years, using competing risk models in a 1:2 propensity score matched cohort.

Table S6. Subdistribution hazard ratios (sHRs) and 95% CIs for the association between use of phentermine and risk of ORCs (BrCa, CRC, ECa, and OCa) among women aged ≥65 years, using competing risk models in a 1:2 propensity score matched cohort.

Table S7. Subdistribution hazard ratios (sHRs) and 95% CIs for the association between use of liraglutide and risk of ORCs (BrCa, CRC, ECa, and OCa) among women aged ≥65 years, using competing risk models in a 1:2 propensity score matched cohort.

Table S1. Variance Inflation Factor for multicollinearity checks among predictors before propensity score matching (n=160,230)

Variable	Degree of Freedom (df)	Variance Inflation Factor (VIF)
Anti-obesity medication	1	1.01525
Age at index date	3	1.22737
Race/ethnicity	3	1.06517
Charlson Comorbidity Index	3	3.56830
Diabetes	1	2.88867
Use of insulin	1	1.13286
Use of metformin	1	1.93631
Hypertension	1	1.34628
Hyperlipidemia	1	1.21953
Cardiovascular disease	1	1.23208
Malaise/fatigue	1	1.07420
Muscular wasting	1	1.00328
Hypogonadism	1	1.00223
Anterior pituitary dysfunction	1	1.00263
Depression	1	1.04315
Osteoporosis	1	1.04086
Cushing's syndrome	1	1.00287
Hypothyroidism	1	1.06829
Hyperthyroidism	1	1.00914
Polycystic ovary syndrome	1	1.00223
Poverty	1	1.10368

VIF <5 indicates low, 5–10 moderate, and >10 high multicollinearity.

Table S2. Baseline characteristics according to the use of phentermine before and after 1:2 propensity score matching.

	No. (%)							
	Pre- 1:2 Propensity Score Matching (n=160,230)				Post- 1:2 Propensity Score Matching (n=6,644)			
**Characteristics**	**Phentermine Never user 158,015 (98.6)**	**Phentermine Ever user 2215 (1.4)**	**p-value**	**% std.diff**	**Phentermine Never user 4430 (66.7)**	**Phentermine Ever user 2214 (33.3)**	**p-value**	**% std.diff**
Age at index date			<0.0001*	0.3087			0.9410	0.0284
• 65 – 70	17,129 (10.8)	383 (17.3)			748 (16.9)	383 (17.3)		
• 70 – 75	38,976 (24.7)	720 (32.5)			1449 (32.7)	720 (32.5)		
• 75 – 80	39,543 (25.0)	505 (22.8)			996 (22.5)	505 (22.8)		
• ≥ 80	62,367 (39.5)	607 (27.4)			1237 (27.9)	606 (27.4)		
Race/ethnicity			<0.0001*	0.2380			0.9711	0.0000
• White	124,367 (78.7)	1,898 (85.7)			3806 (85.9)	1,897 (85.7)		
• Black	15,551 (9.8)	183 (8.3)			368 (8.3)	183 (8.3)		
• Hispanic	4893 (3.1)	63 (2.8)			123 (2.8)	63 (2.8)		
• Other	13,204 (8.4)	71 (3.2)			133 (3.0)	71 (3.2)		
CCI			<0.0001*	0.3092			0.9914	0.0000
• 0	77,337 (48.9)	817 (36.9)			1641 (37.1)	817 (36.9)		
• 1	45,292 (28.7)	644 (29.1)			1272 (28.7)	644 (29.1)		
• 2	20,455 (12.9)	383 (17.3)			771 (17.4)	383 (17.3)		
• 3 or more	14,931 (9.5)	371 (16.8)			746 (16.8)	370 (16.7)		
Diabetes	47,870 (30.3)	454 (20.5)	<0.0001*	-0.2265	917 (20.7)	454 (30.5)	0.8539	-0.0048
Use of insulin	7788 (4.9)	95 (4.3)	0.1669	-0.0305	190 (4.3)	95 (4.3)	0.9971	0.0001
Use of metformin	37,612 (23.8)	220 (9.9)	<0.0001*	-0.3769	404 (9.1)	220 (9.9)	0.2818	0.0278
Hypertension	96,091 (60.8)	1496 (67.5)	<0.0001*	0.1407	2999 (67.7)	1495 (67.5)	0.8871	-0.0037
Hyperlipidemia	67,305 (98.4)	1073 (1.6)	<0.0001*	0.1176	2165 (48.8)	1072 (48.2)	0.7282	-0.0090
CVD	76,543 (48.4)	1339 (60.4)	<0.0001*	0.2430	2688 (60.6)	1338 (60.4)	0.8481	-0.0050
Malaise and fatigue	24,843 (15.7)	662 (29.9)	<0.0001*	0.3425	1330 (30.0)	661 (29.9)	0.8885	-0.0036
Muscular wasting	1554 (0.9)	76 (3.4)	<0.0001*	0.1672	132 (3.0)	76 (3.4)	0.3176	0.0257
Hypogonadism †	28 (0.02)	0 (0.0)	1.0000	-0.0188	<11 (<0.1) ‡	0 (0.0)	1.0000	-0.0213
APD †	94 (0.06)	<11 (<0.2) ‡	0.1513	0.0243	<11 (<0.1) ‡	<11 (<0.2) ‡	0.4071	0.0213
Depression disorder	8896 (5.6)	394 (17.8)	<0.0001*	0.3851	772 (17.4)	393 (17.7)	0.7434	0.0085

Osteoporosis	20,533 (12.9)	398 (17.9)	<0.0001*	0.1378	791 (17.9)	398 (17.9)	0.9035	0.0032
Cushing's syndrome †	41 (0.03)	<11 (<0.1) ‡	0.1191	0.0267	<11 (<0.1) ‡	<11 (<0.1) ‡	0.2591	0.0285
Hypothyroidism	29,264 (18.5)	637 (28.8)	<0.0001*	0.2428	1,297 (29.2)	637 (28.8)	0.6685	-0.0112
Hyperthyroidism	1820 (1.2)	30 (1.4)	0.3754	0.0182	71 (1.6)	30 (1.4)	0.4367	-0.0205
PCOS †	19 (0.01)	<11 (<0.1) ‡	0.2430	0.0196	<11 (<0.1) ‡	<11 (<0.1) ‡	1.0000	-0.0205
Poverty, mean (SD) §	11.5 (8.7)	11.4 (8.4)	0.6485	-0.0096	11.3 (8.1)	11.4 (8.5)	0.4327	0.0203

Abbreviation: APD, anterior pituitary dysfunction; CVD, cardiovascular disease; CCI, Charlson comorbidity index; PCOS, polycystic ovary syndrome; SD, standard deviation; std.diff, standardized difference. * Chi-square statistical significance at the p value < 0.05 level. † Fisher exact test two-sided p-value. ‡ SEER-Medicare data presentation guideline has been followed and all counts less than 11 have been suppressed. § Percent of residents living below poverty.

Table S3. Baseline characteristics according to the use of liraglutide before and after 1:2 propensity score matching.

	No. (%)							
	Pre- 1:2 Propensity Score Matching (n=160,230)				Post- 1:2 Propensity Score Matching (n=807)			
**Characteristics**	**Liraglutide Never user 159,961 (99.8)**	**Liraglutide Ever user 269 (0.2)**	**p-value**	**% std.diff**	**Liraglutide Never user 538 (66.7)**	**Liraglutide Ever user 269 (33.3)**	**p-value**	**% std.diff**
Age at index date			<0.0001*	0.5736			0.9331	0.0537
• 65 – 70	17,441 (10.9)	71 (26.4)			146 (27.1)	71 (26.4)		
• 70 – 75	39,601 (24.8)	95 (35.3)			194 (36.1)	95 (35.3)		
• 75 – 80	39,995 (25.0)	53 (19.7)			108 (20.1)	53 (19.7)		
• ≥ 80	62,924 (39.3)	50 (18.6)			90 (16.7)	50 (18.5)		
Race/ethnicity			0.7292	0.0714			0.1042	0.1830
• White	126,054 (78.8)	>210 (>78.0)			390 (72.5)	>211 (78.4)		
• Black	15,703 (9.8)	31 (11.5)			97 (18.0)	31 (11.5)		
• Hispanic	4948 (3.1)	<11 (<3.0) ‡			19 (3.5)	<11 (<3.0) ‡		
• Other	13,256 (8.3)	19 (7.1)			32 (6.0)	19 (7.1)		
CCI			<0.0001*	1.3023			0.6742	0.0867
• 0	78,146 (48.9)	<11 (<3.0) ‡			15 (2.8)	<11 (<3.0) ‡		
• 1	45,837 (28.7)	>98 (>36.0)			222 (41.3)	>97 (>36.0)		
• 2	20,754 (12.9)	84 (31.2)			153 (28.4)	84 (31.2)		
• 3 or more	15,224 (9.5)	78 (29.0)			148 (27.5)	78 (29.0)		
Diabetes	48,069 (30.1)	255 (94.8)	<0.0001*	1.7973	512 (95.1)	255 (94.8)	0.8186	-0.0170
Use of insulin	7,773 (4.9)	110 (40.8)	<0.0001*	0.9497	215 (39.9)	110 (40.8)	0.7997	0.0189
Use of metformin	37,727 (23.6)	105 (39.0)	<0.0001*	0.3378	214 (39.8)	105 (39.0)	0.8386	-0.0152
Hypertension	97,355 (60.8)	232 (86.3)	<0.0001*	0.6010	443 (82.3)	232 (86.3)	0.1576	0.1074
Hyperlipidemia	68,196 (42.6)	182 (67.6)	<0.0001*	0.5199	348 (64.7)	182 (67.6)	0.4004	0.0629
CVD	77,697 (48.6)	185 (68.8)	<0.0001*	0.4191	369 (68.6)	185 (68.7)	0.9572	0.0040
Malaise and fatigue	25,447 (15.9)	58 (21.6)	0.0113*	0.1453	129 (23.9)	58 (21.6)	0.4431	-0.0576
Muscular wasting †	1626 (1.0)	<11 (<2.0) ‡	0.3574	0.0.423	<11 (<2.0) ‡	<11 (<2.0) ‡	0.7838	-0.0290
Hypogonadism †	28 (0.02)	0 (0.0)	1.0000	-0.0187	0 (0.0)	0 (0.0)	1.0000	0.0000
APD †	97 (0.06)	0 (0.0)	1.0000	-0.0348	<11 (<2.0) ‡	0 (0.0)	1.0000	-0.0610
Depression disorder	9269 (5.8)	21 (7.8)	0.1583	0.0800	40 (7.4)	21 (7.8)	0.8506	0.0140

Osteoporosis	20,901 (13.1)	30 (11.2)	0.3520	-0.0587	60 (11.2)	30 (11.2)	1.0000	0.0000
Cushing's syndrome †	43 (0.03)	0 (0.0)	1.0000	-0.0232	0 (0.0)	0 (0.0)	1.0000	0.0000
Hypothyroidism	29,810 (18.6)	91 (33.8)	<0.0001*	0.3506	178 (33.1)	91 (33.8)	0.8327	0.0158
Hyperthyroidism †	1846 (1.2)	<11 (<2.0) ‡	0.5586	0.0292	<11 (<2.0) ‡	<11 (<2.0) ‡	1.0000	-0.0149
PCOS †	20 (0.01)	0 (0.0)	1.0000	-0.0158	0 (0.0)	0 (0.0)	1.0000	0.0000
Poverty, mean (SD) [c]	11.5 (8.7)	11.3 (8.6)	0.7434	-0.0201	12.2 (9.0)	11.4 (8.7)	0.1920	-0.0981

Abbreviation: APD, anterior pituitary dysfunction; CVD, cardiovascular disease; CCI, Charlson comorbidity index; PCOS, polycystic ovary syndrome; SD, standard deviation; std.diff, standardized difference. * Chi-square statistical significance at the p value < 0.05 level. † Fisher exact test two-sided p-value. ‡ SEER-Medicare data presentation guideline has been followed and all counts less than 11 have been suppressed. § Percent of residents living below poverty.

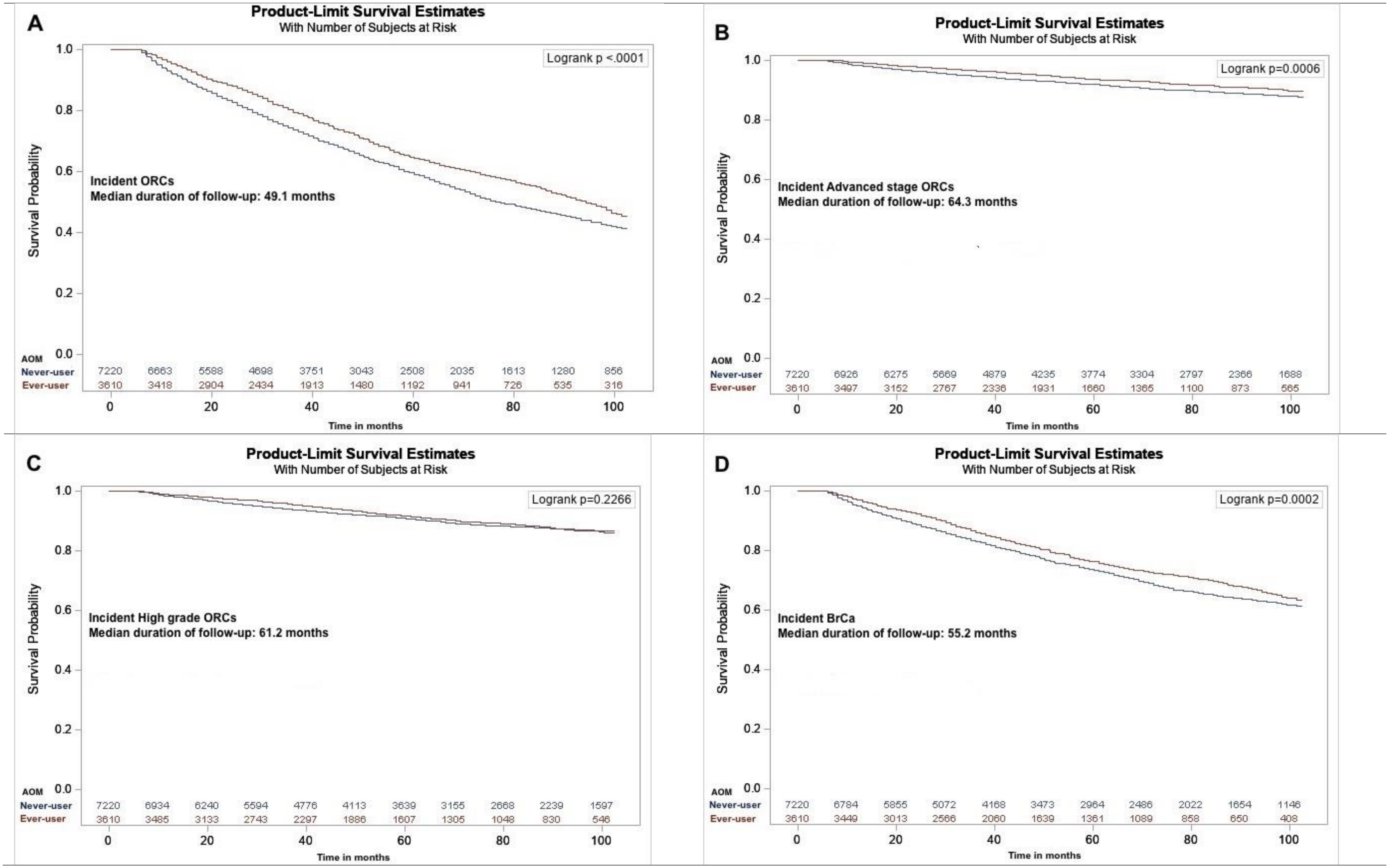
A
Product-Limit Survival Estimates
With Number of Subjects at Risk
Logrank p <.0001
Incident ORCs
Median duration of follow-up: 49.1 months
Survival Probability
AOM
Never-user 7220 6663 5588 4698 3751 3043 2508 2035 1613 1280 856
Ever-user 3610 3418 2904 2434 1913 1480 1192 941 726 535 316
Time in months
B
Product-Limit Survival Estimates
With Number of Subjects at Risk
Logrank p=0.0006
Incident Advanced stage ORCs
Median duration of follow-up: 64.3 months
Survival Probability
AOM
Never-user 7220 6926 6275 5669 4879 4235 3774 3304 2797 2366 1688
Ever-user 3610 3497 3152 2767 2336 1931 1660 1365 1100 873 565
Time in months
C
Product-Limit Survival Estimates
With Number of Subjects at Risk
Logrank p=0.2266
Incident High grade ORCs
Median duration of follow-up: 61.2 months
Survival Probability
AOM
Never-user 7220 6934 6240 5594 4776 4113 3639 3155 2668 2239 1597
Ever-user 3610 3485 3133 2743 2297 1886 1607 1305 1048 830 546
Time in months
D
Product-Limit Survival Estimates
With Number of Subjects at Risk
Logrank p=0.0002
Incident BrCa
Median duration of follow-up: 55.2 months
Survival Probability
AOM
Never-user 7220 6784 5855 5072 4168 3473 2964 2486 2022 1654 1146
Ever-user 3610 3449 3013 2566 2060 1639 1361 1089 858 650 408
Time in months

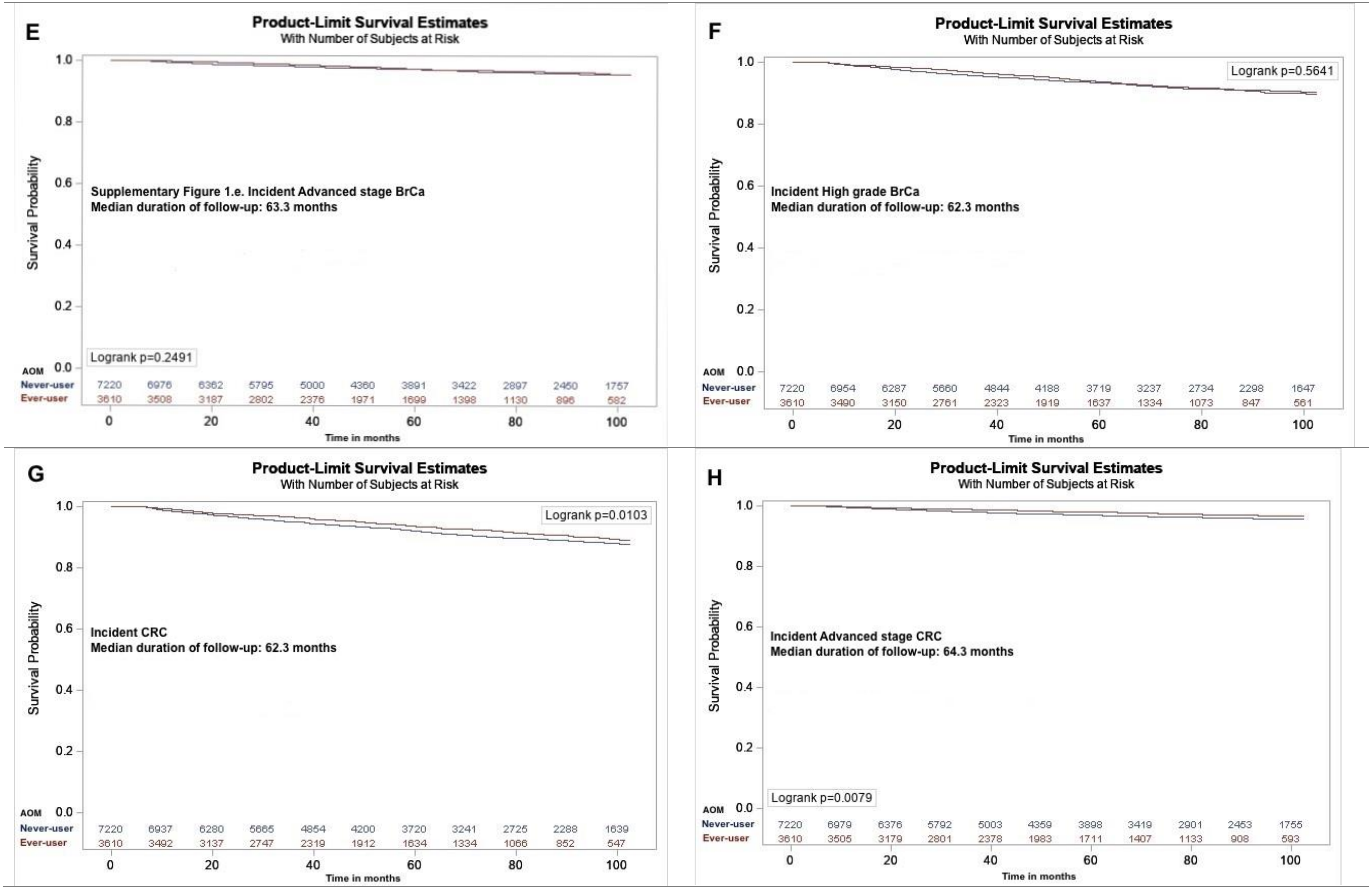
E
Product-Limit Survival Estimates
With Number of Subjects at Risk
Supplementary Figure 1.e. Incident Advanced stage BrCa
Median duration of follow-up: 63.3 months
Logrank p=0.2491
Survival Probability
AOM
Never-user 7220 6976 6362 5795 5000 4360 3891 3422 2897 2450 1757
Ever-user 3610 3508 3187 2802 2376 1971 1699 1398 1130 896 582
Time in months
F
Product-Limit Survival Estimates
With Number of Subjects at Risk
Logrank p=0.5641
Incident High grade BrCa
Median duration of follow-up: 62.3 months
Survival Probability
AOM
Never-user 7220 6954 6287 5660 4844 4188 3719 3237 2734 2298 1647
Ever-user 3610 3490 3150 2761 2323 1919 1637 1334 1073 847 561
Time in months
G
Product-Limit Survival Estimates
With Number of Subjects at Risk
Logrank p=0.0103
Incident CRC
Median duration of follow-up: 62.3 months
Survival Probability
AOM
Never-user 7220 6937 6280 5665 4854 4200 3720 3241 2725 2288 1639
Ever-user 3610 3492 3137 2747 2319 1912 1634 1334 1066 852 547
Time in months
H
Product-Limit Survival Estimates
With Number of Subjects at Risk
Incident Advanced stage CRC
Median duration of follow-up: 64.3 months
Logrank p=0.0079
Survival Probability
AOM
Never-user 7220 6979 6376 5792 5003 4359 3898 3419 2901 2453 1755
Ever-user 3610 3505 3179 2801 2378 1983 1711 1407 1133 908 593
Time in months

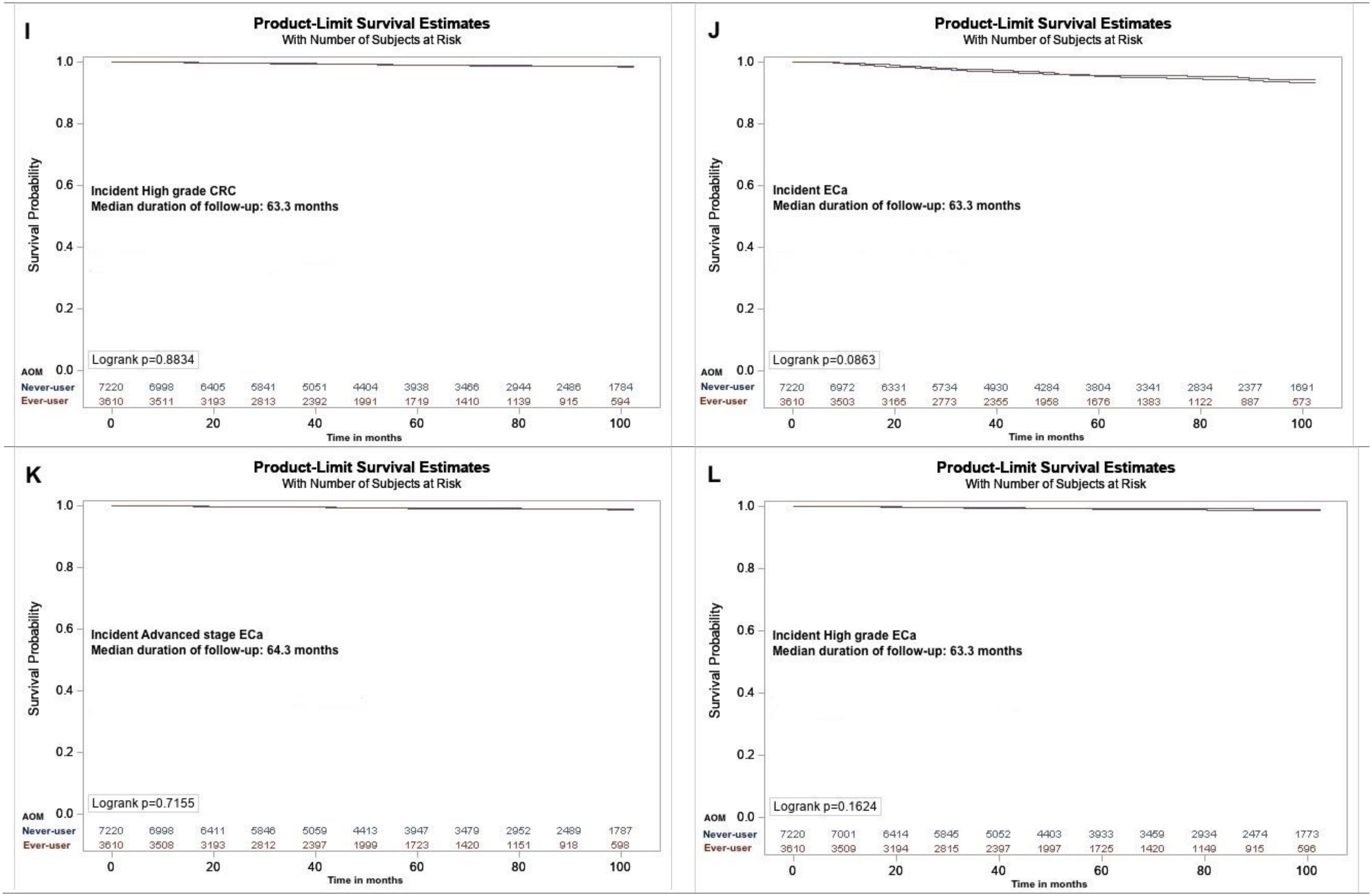
I
Product-Limit Survival Estimates
With Number of Subjects at Risk
Survival Probability
1.0
0.8
0.6
0.4
0.2
0.0
Incident High grade CRC
Median duration of follow-up: 63.3 months
Logrank p=0.8834
AOM
Never-user 7220 6998 6405 5841 5051 4404 3938 3466 2944 2486 1784
Ever-user 3610 3511 3193 2813 2392 1991 1719 1410 1139 915 594
0 20 40 60 80 100
Time in months
J
Product-Limit Survival Estimates
With Number of Subjects at Risk
Survival Probability
1.0
0.8
0.6
0.4
0.2
0.0
Incident ECa
Median duration of follow-up: 63.3 months
Logrank p=0.0863
AOM
Never-user 7220 6972 6331 5734 4930 4284 3804 3341 2834 2377 1691
Ever-user 3610 3503 3165 2773 2355 1958 1676 1383 1122 887 573
0 20 40 60 80 100
Time in months
K
Product-Limit Survival Estimates
With Number of Subjects at Risk
Survival Probability
1.0
0.8
0.6
0.4
0.2
0.0
Incident Advanced stage ECa
Median duration of follow-up: 64.3 months
Logrank p=0.7155
AOM
Never-user 7220 6998 6411 5846 5059 4413 3947 3479 2952 2489 1787
Ever-user 3610 3508 3193 2812 2397 1999 1723 1420 1151 918 598
0 20 40 60 80 100
Time in months
L
Product-Limit Survival Estimates
With Number of Subjects at Risk
Survival Probability
1.0
0.8
0.6
0.4
0.2
0.0
Incident High grade ECa
Median duration of follow-up: 63.3 months
Logrank p=0.1624
AOM
Never-user 7220 7001 6414 5845 5052 4403 3933 3459 2934 2474 1773
Ever-user 3610 3509 3194 2815 2397 1997 1725 1420 1149 915 596
0 20 40 60 80 100
Time in months

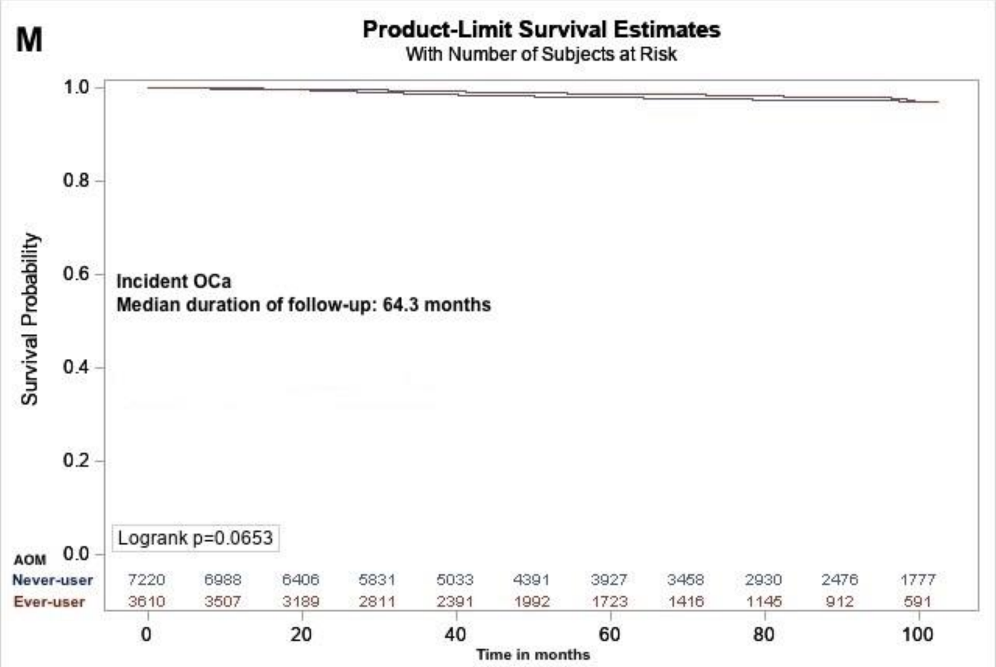

Figure S1. Non-parametric Kaplan–Meier estimates of time to incident ORCs stratified by the pre-diagnostic use of AOM in 1:2 propensity score matched cohort. Censoring information (hashmarks) have been suppressed according to SEER-Medicare data presentation guideline. **(A)** Incident ORC; **(B)** Incident Advanced stage ORC; **(C)** Incident High grade ORC; **(D)** Incident BrCa; **(E)** Incident Advanced stage BrCa; **(F)** Incident High grade BrCa; **(G)** Incident CRC; **(H)** Incident Advanced stage CRC; **(I)** Incident High grade CRC; **(J)** Incident ECa; **(K)** Incident Advanced stage ECa; **(L)** Incident High grade ECa; **(M)** Incident OCa; Abbreviations: aHR, adjusted hazard ratio; AOM, anti-obesity medication; BrCa, breast cancer; CRC, colorectal cancer; ECa, endometrial cancer; OCa, ovarian cancer; ORCs, obesity-related cancers. AOM includes diethylpropion, liraglutide, lorcaserin, orlistat, phendimetrazine and phentermine. Time-to-event was calculated in months from the index date (first AOM exposure). For patients who developed an ORCs, time-to-event was measured from the index date to the first ORC diagnosis. Patients who did not develop an ORCs were right-censored at the study end date (December 31, 2015) or at the time of loss to follow-up. Patients who were ORCs-free and died before the study end date were right-censored at the time of death.

Table S4. HRs and 95% CIs for the association between use of AOM and risk of ORCs (BrCa, CRC, ECa, and OCa) among women aged ≥65 years with and without diabetes, in a 1:2 propensity score matched cohort.

	**Without Diabetes**		**With Diabetes**	
**Category**	Adjusted HR (95% CI)	p-value	Adjusted HR (95% CI)	p-value
ORCs incident				
• AOM never-user	Ref		Ref	
• AOM ever-user	0.78 (0.71 , 0.85)	<0.0001*	0.81 (0.73 , 0.89)	<0.0001*
Advanced stage ORCs incident				
• AOM never-user	Ref		Ref	
• AOM ever-user	0.88 (0.70 , 1.09)	0.2331	0.66 (0.53 , 0.83)	0.0003*
High grade ORCs incident				
• AOM never-user	Ref		Ref	
• AOM ever-user	0.93 (0.84 , 1.22)	0.9294	0.83 (0.68 , 1.02)	0.0786
BrCa incident				
• AOM never-user	Ref		Ref	
• AOM ever-user	0.81 (0.73 , 0.92)	0.0007*	0.88 (0.78 , 0.99)	0.0459*
Advanced stage BrCa incident				
• AOM never-user	Ref		Ref	
• AOM ever-user	0.96 (0.68 , 1.36)	0.8377	0.76 (0.53 , 1.10)	0.1535
High grade BrCa incident				
• AOM never-user	Ref		Ref	
• AOM ever-user	0.98 (0.79 , 1.23)	0.9003	0.93 (0.73 , 1.17)	0.5288
CRC incident				
• AOM never-user	Ref		Ref	
• AOM ever-user	0.88 (0.71 , 1.10)	0.2694	0.77 (0.62 , 0.95)	0.0157*
Advanced stage CRC incident				
• AOM never-user	Ref		Ref	
• AOM ever-user	0.75 (0.51 , 1.11)	0.1488	0.66 (0.46 , 0.94)	0.0230*
High grade CRC incident				
• AOM never-user	Ref		Ref	
• AOM ever-user	1.60 (0.96 , 2.66)	0.0710	0.49 (0.23 , 1.06)	0.0692
ECa incident				
• AOM never-user	Ref		Ref	
• AOM ever-user	0.73 (0.53 , 1.01)	0.0547	0.91 (0.69 , 1.19)	0.4759
Advanced stage ECa incident				
• AOM never-user	Ref		Ref	
• AOM ever-user	0.86 (0.44 , 1.67)	0.6508	0.97 (0.49 , 1.89)	0.9339
High grade ECa incident				
• AOM never-user	Ref		Ref	
• AOM ever-user	0.68 (0.33 , 1.41)	0.3114	0.73 (0.39 , 1.35)	0.3116
OCa incident				
• AOM never-user	Ref		Ref	
• AOM ever-user	0.98 (0.66 , 1.47)	0.9474	0.49 (0.28 , 0.86)	0.0120*

Cox models adjusted for age at index date after propensity score matching. AOM includes diethylpropion, liraglutide, lorcaserin, orlistat, phendimetrazine and phentermine. *Statistical

significance at the p value < .05 level. Abbreviations: AOM, anti-obesity medication; BrCa, breast cancer; CI, confidence interval; CRC, colorectal cancer; ECa, endometrial cancer; HR, hazard ratio; OCa, ovarian cancer; ORCs, obesity-related cancers.

Table S5. Subdistribution hazard ratios (sHRs) and 95% CIs for the association between use of AOM and risk of ORCs (BrCa, CRC, ECa, and OCa) among women aged ≥65 years, using competing risk models in a 1:2 propensity score matched cohort.

Category	Adjusted sHR (95% CI)	p-value
ORCs incident		
• AOM never-user	Ref	
• AOM ever-user	0.78 (0.74 , 0.84)	<0.0001*
Advanced stage ORCs incident		
• AOM never-user	Ref	
• AOM ever-user	0.76 (0.59 , 0.89)	0.0004*
High grade ORCs incident		
• AOM never-user	Ref	
• AOM ever-user	0.92 (0.79 , 1.05)	0.2153
BrCa incident		
• AOM never-user	Ref	
• AOM ever-user	0.85 (0.78 , 0.92)	<0.0001*
Advanced stage BrCa incident		
• AOM never-user	Ref	
• AOM ever-user	0.86 (0.67 , 1.11)	0.2439
High grade BrCa incident		
• AOM never-user	Ref	
• AOM ever-user	0.96 (0.81 , 1.12)	0.5718
CRC incident		
• AOM never-user	Ref	
• AOM ever-user	0.82 (0.69 , 0.95)	0.0088*
Advanced stage CRC incident		
• AOM never-user	Ref	
• AOM ever-user	0.69 (0.53 , 0.91)	0.0082*
High grade CRC incident		
• AOM never-user	Ref	
• AOM ever-user	1.03 (0.68 , 1.57)	0.8748
ECa incident		
• AOM never-user	Ref	
• AOM ever-user	0.83 (0.67 , 1.02)	0.0793
Advanced stage ECa incident		
• AOM never-user	Ref	
• AOM ever-user	0.91 (0.57 , 1.45)	0.6910
High grade ECa incident		
• AOM never-user	Ref	
• AOM ever-user	0.71 (0.45 , 1.14)	0.1609
OCa incident		
• AOM never-user	Ref	
• AOM ever-user	0.74 (0.53 , 1.02)	0.0643

Fine-Gray competing risk models adjusted for age at index date after propensity score matching. All-cause death was treated as the competing event for ORCs. AOM includes diethylpropion, liraglutide, lorcaserin, orlistat, phendimetrazine and phentermine. *Statistical significance at the p

value < .05 level. Abbreviations: AOM, anti-obesity medication; BrCa, breast cancer; CI, confidence interval; CRC, colorectal cancer; ECa, endometrial cancer; OCa, ovarian cancer; ORCs, obesity-related cancers; sHR; subdistribution hazard ratio.

Table S6. Subdistribution hazard ratios (sHRs) and 95% CIs for the association between use of phentermine and risk of ORCs (BrCa, CRC, ECa, and OCa) among women aged ≥65 years, using competing risk models in a 1:2 propensity score matched cohort.

Category	Adjusted sHR (95% CI)	p-value
ORCs incident		
• Phentermine never-user	Ref	
• Phentermine ever-user	0.71 (0.65 , 0.77)	<0.0001*
Advanced stage ORCs incident		
• Phentermine never-user	Ref	
• Phentermine ever-user	0.96 (0.77 , 1.18)	0.6674
High grade ORCs incident		
• Phentermine never-user	Ref	
• Phentermine ever-user	1.05 (0.87 , 1.27)	0.5973
BrCa incident		
• Phentermine never-user	Ref	
• Phentermine ever-user	0.73 (0.65 , 0.81)	<0.0001*
Advanced stage BrCa incident		
• Phentermine never-user	Ref	
• Phentermine ever-user	0.88 (0.61 , 1.26)	0.4849
High grade BrCa incident		
• Phentermine never-user	Ref	
• Phentermine ever-user	1.02 (0.83 , 1.27)	0.8272
CRC incident		
• Phentermine never-user	Ref	
• Phentermine ever-user	0.86 (0.69 , 1.06)	0.1516
Advanced stage CRC incident		
• Phentermine never-user	Ref	
• Phentermine ever-user	0.87 (0.61 , 1.23)	0.4343
High grade CRC incident		
• Phentermine never-user	Ref	
• Phentermine ever-user	1.05 (0.64 , 1.71)	0.8527
ECa incident		
• Phentermine never-user	Ref	
• Phentermine ever-user	0.71 (0.53 , 0.95)	0.0234*
Advanced stage ECa incident		
• Phentermine never-user	Ref	
• Phentermine ever-user	1.09 (0.58 , 2.05)	0.7987
High grade ECa incident		
• Phentermine never-user	Ref	
• Phentermine ever-user	0.99 (0.48 , 2.09)	0.9982
OCa incident		
• Phentermine never-user	Ref	
• Phentermine ever-user	1.09 (0.72 , 1.66)	0.6833

Fine-Gray competing risk models adjusted for age at index date after propensity score matching. All-cause death was treated as the competing event for ORCs. *Statistical significance at the p value < .05 level. Abbreviations: BrCa, breast cancer; CI, confidence interval; CRC, colorectal cancer; ECa,

endometrial cancer; OCa, ovarian cancer; ORCs, obesity-related cancers; sHR; subdistribution hazard ratio.

Table S7. Subdistribution hazard ratios (sHRs) and 95% CIs for the association between use of liraglutide and risk of ORCs (BrCa, CRC, ECa, and OCa) among women aged ≥65 years, using competing risk models in a 1:2 propensity score matched cohort.

Category	Adjusted sHR (95% CI)	p-value
ORCs incident		
• Liraglutide never-user	Ref	
• Liraglutide ever-user	0.71 (0.54 , 0.93)	0.0141*
Advanced stage ORCs incident		
• Liraglutide never-user	Ref	
• Liraglutide ever-user	0.68 (0.36 , 1.29)	0.2377
High grade ORCs incident		
• Liraglutide never-user	Ref	
• Liraglutide ever-user	0.94 (0.53 , 1.66)	0.8216
BrCa incident		
• Liraglutide never-user	Ref	
• Liraglutide ever-user	0.78 (0.56 , 1.10)	0.1624
Advanced stage BrCa incident		
• Liraglutide never-user	Ref	
• Liraglutide ever-user	1.19 (0.40 , 3.58)	0.7470
High grade BrCa incident		
• Liraglutide never-user	Ref	
• Liraglutide ever-user	1.09 (0.55 , 2.16)	0.8058
CRC incident		
• Liraglutide never-user	Ref	
• Liraglutide ever-user	0.52 (0.26 , 1.08)	0.0778
Advanced stage CRC incident		
• Liraglutide never-user	Ref	
• Liraglutide ever-user	0.41 (0.12 , 1.40)	0.1543
High grade CRC incident		
• Liraglutide never-user	Ref	
• Liraglutide ever-user	NC	NC
ECa incident		
• AOM never-user	Ref	
• AOM ever-user	1.31 (0.72 , 2.39)	0.3735
Advanced stage ECa incident		
• AOM never-user	Ref	
• AOM ever-user	1.03 (0.32 , 3.32)	0.9655
High grade ECa incident		
• AOM never-user	Ref	
• AOM ever-user	1.14 (0.29 , 4.34)	0.8488
OCa incident		
• Liraglutide never-user	Ref	
• Liraglutide ever-user	0.25 (0.03 , 1.87)	0.1744

Fine-Gray competing risk models adjusted for age at index date after propensity score matching. All-cause death was treated as the competing event for ORCs. Abbreviations: BrCa, breast cancer;

CI, confidence interval; CRC, colorectal cancer; ECa, endometrial cancer; NC; not calculated; OCa, ovarian cancer; ORCs, obesity-related cancers; sHR; subdistribution hazard ratio
